# Advances in Nanomaterials for Lithium-Ion/Post-Lithium-Ion Batteries and Supercapacitors

**DOI:** 10.3390/nano12152512

**Published:** 2022-07-22

**Authors:** Mario Marinaro, Sonia Dsoke

**Affiliations:** 1 ZSW-Zentrum für Sonnenenergie und Wasserstoff-Forschung, Helmholtzstrasse 8, 89081 Ulm, Germany; 2 Institute for Applied Materials (IAM), Karlsruhe Institute of Technology (KIT), Hermann-von-Helmholtz-Platz 1, 76344 Eggenstein-Leopoldshafen, Germany

Energy storage and conversion are key factors for enabling the transition from fossil fuels to intermittent renewables. Moreover, the increasing number of electric vehicles on the road (HEV, PHEV, BEV, FCEV) calls for high-performance, reliable, safe, and low-cost energy storage and conversion devices. Within this context, batteries and supercapacitors as well as hydrogen and fuel cells play a central role. Our point of view is that depending on the application, all storage and conversion devices should contribute to the final goal of decreasing greenhouse gas emissions by speeding up the electrification process.

Lithium-ion batteries (LIBs) are paving the way as the technology of choice for the electrification of road transport as well as for home storage. Since being commercialized by Sony in 1991, the cost of this technology has decreased from USD ~1000 to ~100 per kW/h, representing approximately one order of magnitude. At the same time, achievable energies have improved substantially. Increasing battery energy not only means developing new high-performance anode and cathode materials, but also means improving the performance and minimizing the weight and volume fractions of all of the inactive components.

Besides LIBs, there are new and emerging technologies, so-called “post Lithium-ion” batteries, such as Na-ion, solid-state, and Mg- and Ca-ion batteries, which, if successfully developed, might introduce some advantages. It is worth noting that the abovementioned battery technologies are in different stages of development and therefore have different Technology Readiness Levels (TRLs), with Na-ion batteries being the closest to market introduction.

This Special Issue focuses on the current developments and future perspectives of various battery and supercapacitor technologies through eight original research papers and one review, all of which were written by well-respected scientists.

As already mentioned, Na-ion batteries are the post-lithium battery technology that is the closest to market introduction. In fact, Na-ion is often referred to as drop-in technology, as it may not require the full re-organization of the battery cell manufacturing process. Mosa and co-workers report on the electrochemical conversion reaction of NiO with Na-ions in an organic electrolyte [1]. NiO thin films (560 nm thick) are successfully prepared by magnetron sputtering (MS) deposition under an oblique angle configuration (OAD) and demonstrate a large capacity during the first cycle that then fades remarkably upon successive cycles, demonstrating the limited reversibility of the electrochemically driven conversion reaction.

Bargnesi et al. still focus Na-ion batteries, this time with an aqueous electrolyte, and suggest the use of cross-linked chitosan as a binder for an environmentally friendly electrode preparation process [2]. A successful cross-linked reaction involving chitosan and succinic acid with the addition of a coupling agent, N-(3-Dimethylaminopropyl)-N′-ethylcarbodiimide hydrochloride, resulted in a water-insoluble binder that allows the manufacturing of free-standing electrodes for aqueous Na-ion batteries.

Considering solid-state batteries, Yang and co-workers propose a solid electrolyte based on Li_2_(BH_4_)(NH_2_) that was nanoconfined in a mesoporous silica molecule sieve (SBA-15) and obtained via the melting–infiltration approach [3]. The SBA-15-modified electrolyte shows improved Li-ion conductivity over the unmodified Li_2_(BH_4_)(NH_2_). The authors successfully demonstrate low-voltage proof-of-concept ASSBs using TiS_2_ as a cathode material capable of achieving ~50 cycles at T = 55 °C.

Silicon and silicon oxides are a very promising class of anode material for LIBs, as they can reach a specific capacity that is up to 10× higher compared to state-of-the-art graphite. Tenzg and co-workers proposed a silicon carbide (SiC)/graphitic carbon coating made of silicon flakes recycled from the waste produced from silicon wafers [4]. The coated material showed improved electrochemical performance in half cells. The authors ascribe the improved performance to the double effect of SiC, which provides physical strength and helps to maintain the integrity of the silicon flakes and to isolate silicon from irreversible reactions with the electrolytes. Moreover, the carbon coating provides enhanced electrical conductivity for the SiC-encapsulated silicon flakes.

Magnesium-based batteries represent one of the more successful electrochemical energy storage chemistries that has been emerging. Magnesium is abundant in the Earth and, due to its high density, can provide a volumetric capacity higher than lithium (3850 vs. 2046 mAh·cm^−3^). The review proposed by Bella and co-workers [5] focuses on the latest developments in anode materials for Mg-ion batteries. On the one hand, the manuscript offers insights into the challenges and, on the other, provides guidance on how they may be addressed in order to further develop this battery technology.

Materials that can be applied to supercapacitors can be classified into double-layer and faradaic types. Faradaic materials can be further distinguished as batteries and pseudocapacitive types depending on their electrochemical signature. So-called “pseudocapacitive” materials are compounds that, even though they undergo redox reactions, display an electrochemical signature that is similar to that of a double-layer-type material (i.e., cyclic voltammetry with a box-type shape). There are few materials with this particular property, with the most prominent examples being MnO_2_ and RuO_2_. In the framework of this Special Issue, a needle-like MnO*_x_*@graphite nanostructure has been proposed by Ray et al. [6]. This nanostructured electrode was used in a symmetric pouch cell configuration with a solid-state polymer gel electrolyte, showing an encouraging performance (areal energy density (Ea) of 7.87 mWh·cm^−2^ and areal power density (Pa) of 1099.64 mW·cm^−2^ with a 2.2.V cell voltage).

Thierry Brousse’s group [7] proposed a novel material, Fe_2_WO_6_, which can be added to the short list of compounds exhibiting pseudocapacitive behavior. When used in an aqueous electrolyte as a negative electrode, Fe_2_WO_6_ can be combined with MnO_2_-positive electrode material, thus resulting in a device with an enhanced energy density with respect to classical EDLCs based on carbon electrodes.

In addition to the pseudocapacitive materials, high-power faradaic compounds with clear redox peaks can be used in an asymmetric configuration, where a battery-type electrode is coupled with a double-layer-type one. Sharma et al. [8] explored NiMoO_4_ and NiWO_4_ as positive electrode materials and investigated the effects of their anionic counterparts (MoO_4_^−^ and NiWO_4_^−^) on the electrochemical properties. They found that the redox reaction of NiMoO_4_ only involves a reaction between Ni^2+^ and Ni^3+^ at the surface, while NiWO_4_ shows a two-step redox reaction that includes the participation of a tungsten oxide layer via a bulk-type reaction. Among these two materials, NiMoO_4_ is considered to be the favorite candidate for supercapacitor applications.

The crucial role of inactive components, such as binders, is highlighted by the work of Bargnesi et al., who implement a chitosan binder for both Na-ion batteries and supercapacitor-type electrodes based on activated carbon [2].

A further step that would enhance the overall energy density is to find strategies for the elimination of binders from electrodes also on a large-scale production. In this respect, Ahmed et al. [9] grew ZnO nanorods directly on an aluminum substrate and applied them as a high-power electrode. This electrode material is promising for use in hybrid supercapacitors due to its high rate capability and stability over 1000 cycles.

In summary, this Special Issue compiles a series of original research articles and one review paper, providing new insights and advances in materials and components for post-lithium systems and high-power devices, and it will be of great interest to the readers of *Nanomaterials*.
